# Supplementary material for: Seed treatment using methyl jasmonate induces resistance to rice water weevil but reduces plant growth in rice
Source: PLoS One. 2019 Sep 23;14(9):e0222800. doi: 10.1371/journal.pone.0222800 (PMC6756538; doi:10.1371/journal.pone.0222800)
Supplement: S1 Table — Dates of agricultural practices including seed treatments, water seeding, flooding, and foliar sprays. Sample dates for emergence, biomass, rice water weevil core samples, heading, and harvest. (PDF) [file pone.0222800.s001.pdf]

Experiment	Seed Treatment	Water Seeding	Emergence	Flooding	Foliar Sprays	Biomass	Core Sampling	Heading	Harvest
2017	21-Apr	23-Apr	5-May	17-May	15-May	18-May	23-May	12-Jul	18-Aug
			8-May		19-May	26-May	31-May	14-Jul	
					24-May	31-May	7-Jun	19-Jul	
					31-May	7-Jun	14-Jun		
						14-Jun	21-Jun		
2018	25-Mar	27-Mar	9-Apr	4-May	4-May	8-May	22-May	6-Jul	6-Aug
			19-Apr		8-May	22-May	1-Jun	13-Jul	
					11-May				
					14-May				
					17-May				
