# Supplementary material for: Seed treatment using methyl jasmonate induces resistance to rice water weevil but reduces plant growth in rice
Source: PLoS One. 2019 Sep 23;14(9):e0222800. doi: 10.1371/journal.pone.0222800 (PMC6756538; doi:10.1371/journal.pone.0222800)
Supplement: S2 Table — Mean and standard error are provided for each treatment, on each sample date, for all response variables in experiments in 2017 and 2018. (PDF) [file pone.0222800.s002.pdf]

Response Variable	Year	Sample Date	Treatment	Mean±SE	Notes
Densities of RWW	2017	1	Control	0.5±0.25	
			Karate	0.19±0.13	
			MJ	0.06±0.06	
			MJ+Karate	0.06±0.06	
		2	Control	5.0 ± 1.85	
			Karate	2.64± 0.71	
			MJ	3.21±0.86	
			MJ+Karate	2.38 ± 0.71	
		3	Control	9.13 ±3.14	
			Karate	2.56 ± 0.95	
			MJ	5.56 ± 1.30	
			MJ+Karate	3.5 ± 1.56	
		4	Control	15.43 ± 1.75	
			Karate	4.63 ± 0.60	
			MJ	8.5 ± 2.21	
			MJ+Karate	5.5 ± 1.4	
		5	Control	16.31 ± 3.48	
			Karate	12.25 ± 2.10	
			MJ	14.06 ± 2.17	
			MJ+Karate	8.38 ± 1.29	
	2018	1	Control	0.01	Excluded from analysis
			Karate	0	Excluded from analysis
			MJ	0	Excluded from analysis
			MJ+Karate	0	Excluded from analysis
		2	Control	6 ±1.88	
			Karate	1.63 ± 0.38	
			MJ	4.56 ± 1.14	
			MJ+Karate	1.75 ± 0.59	
Plant Emergence	2017	1	Control	41.44 ± 3.15	
			MJ	21.41 ± 2.03	
		2	Control	43.06 ± 4.54	
			MJ	23.0 ± 2.31	
	2018	1	Control	22.06 ± 1.17	
			MJ	8.38 ± 0.89	
		2	Control	23.56 ± 1.59	
			MJ	10.56 ± 1.38	
Plant Biomass (Root)	2017	1	Control	15.46 ± 1.41	
			Karate	12.29 ± 1.63	
			MJ	11.15 ± 1.13	

			MJ+Karate	13.71 ± 0.65	
		2	Control	37.16 ± 5.52	
			Karate	41.64 ± 5.12	
			MJ	27.44 ± 2.18	
			MJ+Karate	26.51 ± 2.48	
		3	Control	55.58 ± 6.16	
			Karate	87.11 ± 12.96	
			MJ	48.33 ± 7.04	
			MJ+Karate	55.1 ± 2.29	
		4	Control	104.69 ± 15.04	
			Karate	135.13 ± 18.58	
			MJ	76.0 ± 6.00	
			MJ+Karate	90.5 ± 7.71	
		5	Control	369.50 ± 79.27	
			Karate	1100.75 ± 584.86	
			MJ	222.0 ± 36.48	
			MJ+Karate	471.50 ± 161.38	
Plant Biomass (Shoot)	2017	1	Control	27.68 ± 2.0	
			Karate	26.70 ± 2.85	
			MJ	18.32 ± 0.92	
			MJ+Karate	22.33 ± 1.44	
		2	Control	109.74 ± 17.48	
			Karate	87.21 ± 10.48	
			MJ	55.55 ± 5.20	
			MJ+Karate	58.07 ± 3.79	
		3	Control	116.30 ± 12.99	
			Karate	206.54 ± 36.96	
			MJ	105.81 ± 17.27	
			MJ+Karate	123.0 ± 6.08	
		4	Control	310.19 ± 47.50	
			Karate	355.38 ± 43.12	
			MJ	229.06 ± 10.32	
			MJ+Karate	262.25 ± 27.61	
		5	Control	735.06 ± 125.18	
			Karate	873.63 ± 156.81	
			MJ	576.13 ± 129.16	
			MJ+Karate	796.56 ± 181.26	
Plant Biomass (Root)	2018	1	Control	26.35 ± 1.84	
			Karate	26.50 ± 2.27	
			MJ	21.36 ± 1.86	
			MJ+Karate	19.64 ± 3.96	

		2	Control	176.60 ± 14.73	
			Karate	138.69 ± 10.69	
			MJ	152.44 ± 17.6	
			MJ+Karate	174.05 ± 22.70	
Plant Biomass (Shoot)	2018	1	Control	65.93 ± 3.87	
			Karate	69.76 ± 6.78	
			MJ	58.02 ± 3.91	
			MJ+Karate	43.53 ± 4.84	
		2	Control	356.48 ± 35.71	
			Karate	303.97 ± 27.93	
			MJ	306.05 ± 42.13	
			MJ+Karate	352.15 ± 35.13	
Panicle Densities	2017	1	Control	1.13 ± 0.99	
			Karate	3.31 ±1.48	
			MJ	0.06 ±0	
			MJ+Karate	0.19 ± 0.13	
		2	Control	5.31 ± 3.32	
			Karate	23.44 ± 6.38	
			MJ	0.06 ± 0.06	
			MJ+Karate	0.94 ± 0.49	
		3	Control	42.75 ± 9.64	
			Karate	96.25 ± 12.78	
			MJ	0.38 ± 0.26	
			MJ+Karate	31.75 ± 8.9	
	2018	1	Control	44.88 ± 7.94	
			Karate	42.13 ± 5.95	
			MJ	13.38 ± 3.13	
			MJ+Karate	10.63 ± 3.20	
		2	Control	118.63 ± 11.71	
			Karate	132.63 ± 5.47	
			MJ	76.5 ± 8.87	
			MJ+Karate	77.5 ± 6.3	
Grain Yields (per panicle)	2017	1	Control	1.24 ± 0.15	
			Karate	1.96 ± 0.15	
			MJ	1.22 ± 0.14	
			MJ+Karate	1.80 ± 0.22	
	2018	1	Control	2.7 ± 0.08	
			Karate	2.95 0.08	
			MJ	2.33 ± 0.17	
			MJ+Karate	2.71 ± 0.15	

Grain Yield (per plant)	2018	1	Control	10.0 ± 0.68	
			Karate	10.64 ± 0.55	
			MJ	9.42 ± 1.18	
			MJ+Karate	10.08 ± 0.94	
