# Supplementary material for: Seed treatment using methyl jasmonate induces resistance to rice water weevil but reduces plant growth in rice
Source: PLoS One. 2019 Sep 23;14(9):e0222800. doi: 10.1371/journal.pone.0222800 (PMC6756538; doi:10.1371/journal.pone.0222800)
Supplement: Attachment — Submitted filename: Response to reviewer.docx [file pone.0222800.s003.docx]

5. Review Comments to the Author

I have just some minor concerns:

Line 301, “The effect of time was also significant”, there is no data to prove this. May be it can be put in the supplementary materials.

A supplementary table was created which includes the mean and standard error for each treatment on each individual sampling date. (Line 303)

Line 315, “On the second sampling date, densities of RWW immatures were 73% lower in Karate treated plots than in control plots”. There was also no data to support this.

A supplementary table was created which includes the mean and standard error for each treatment on each individual sampling date. (Line 317)

Line 322-325, “Three days later, numbers of plants emerged were approximately 10% higher overall” “There was no interaction between MJ and time”. No data support.

A supplementary table was created which includes the mean and standard error for each treatment on each individual sampling date. (Lines 325-326)

Line 331-332,334-335, 338-339, 351-352, 367-368, 376-377, 389-390, 407-412, no data support. Line 355-358,365-367, no data support. Is it possible to put some supplemental tables or figures?

Figure 4 contains much of this data, but was not noted in the text. This has been corrected. Additionally a supplementary table was created which includes the mean and standard error for each treatment on each individual sampling date. (Lines 333-334, 337, 341, 345, 354, 356, 357, 361, 365-366, 370-371, 380,393,415)

Reviewer #2:

I have only a minor suggestion: In the title, “an herbivore” could be replaced by “rice water weevil”.

This suggestion was included and the title altered
